# Growth performance and immune response of broilers during active *Eimeria* infection are modified by dietary inclusion of canola meal or corn-DDGS in reduced-protein corn-soybean meal diets

**DOI:** 10.1016/j.aninu.2024.05.007

**Published:** 2024-08-07

**Authors:** Revathi Shanmugasundaram, Adeleye M. Ajao, Shahna Fathima, Adelumola Oladeinde, Ramesh K. Selvaraj, Todd J. Applegate, Oluyinka A. Olukosi

**Affiliations:** a Toxicology and Mycotoxin Research Unit, USDA-ARS, Athens, GA, USA; b Department of Poultry Science, University of Georgia, Athens, GA, USA; c Egg and Poultry Production Safety Research Unit, USDA-ARS, Athens, GA, USA

**Keywords:** Reduced-protein diet, *Eimeria*, Broiler chicken, Growth, Immune response

## Abstract

The objective of this experiment was to study the effects of partial replacement of soybean meal (SBM) with canola meal (CM) or corn-distillers' dried grains with solubles (cDDGS) in reduced-protein (RP) diets for *Eimeria*-infected broilers. A total of 1120 broiler chicks were distributed in a 4 × 2 (4 diets × with or without infection) factorial arrangement with 7 replicates per treatment and 20 birds per replicate. The 4 diets, fed between d 7 and 42, were (i) a standard diet with crude protein at 200 g/kg (SP); (ii) a RP (crude protein at 160 g/kg) corn-SBM diet (RP-SBM); (iii) a RP diet in which 80 g/kg CM replaced 60 g/kg SBM (RP-CM); and (iv) a RP diet in which 100 g/kg cDDGS replaced 50 g/kg SBM (RP-cDDGS). On d 15, birds were infected with mixed *Eimeria* (+E) oocysts. Birds and feed were weighed at intervals for growth performance, and samples for immunology responses were collected on d 21. The results showed as follows: 1) during the acute infection phase, diet × *Eimeria* infection was shown by the diets having no effect in the uninfected group. In contrast, the RP-SBM diet tended to produce higher (*P* < 0.10) weight gain among the infected birds. The d 42 body weight was greater (*P* = 0.001) for the uninfected birds. 2) There was a significant diet × *Eimeria* infection on bile anti-*Eimeria* immunoglobulin A (IgA) concentrations (*P* = 0.015), splenocyte proliferation, macrophage nitric oxide (NO) production (*P* < 0.001), and cecal tonsil interleukin (*IL*)-17 mRNA amounts (*P* < 0.001). Most of these responses were not influenced by the diets in the uninfected birds. However, among the infected birds, birds fed RP-SBM had higher (*P* < 0.05) bile IgA than those fed SP or RP-cDDGS. For the spleen, the interaction was that birds fed RP-SBM or RP-cDDGS diets had the highest or lowest NO production, respectively, and birds that received RP-SBM had greater (*P* < 0.05) splenic CD^8+^:CD^4+^ cell ratio than other diets. In conclusion, partial replacement of SBM with CM or cDDGS had only a marginal effect on d 42 body weight and FCR of the broiler chickens receiving the RP diets. In contrast, these had a negative impact on the immune responses of the broiler chickens.

## 1 Introduction

Coccidiosis is an enteric disease of poultry, primarily caused by the apicomplexan parasite *Eimeria*, with significant economic implications for the global poultry industry (Lillehoj and Lillehoj, 2000; Shirley et al., 2007). The pathogenicity of *Eimeria* involves damage to the intestinal epithelium, inflammation, and immune system activation through altering interleukin (IL)-10 and IL-17 cytokine production (Lillehoj, 1998; Kim et al., 2019; Fathima and Selvaraj, 2022). This ultimately compromises intestinal integrity, reduces digestion and absorption, decreases production performance, increases susceptibility to secondary bacterial infections, and can result in mortality. Traditional control strategies for coccidiosis have heavily relied on the widespread use of chemotherapeutic agents, leading to the emergence of drug resistance (Fathima and Selvaraj, 2022; Ghafouri et al., 2023). Although vaccination has proven effective in preventing the disease, it has several challenges, including cost-effectiveness, geographic strain variations, reduced early growth performance, and increased susceptibility of chicks to secondary bacterial infections (Cai et al., 2022; Liu et al., 2023). Therefore, exploring alternative approaches to control or mitigate the impact of coccidiosis in poultry is critical.

The primary aim of the current experiment was to explore the impact of partly replacing soybean meal (SBM) with canola meal (CM) or corn distillers' dried grains with solubles (cDDGS) in reduced-protein (RP) diets using growth performance and immune responses during the active phase of a mixed *Eimeria* challenge as response criteria. Nutrition plays a pivotal role in the development of coccidiosis in poultry, significantly influencing susceptibility to disease, disease progression, post-infection recovery, and compensatory growth (Gomez-Osorio et al., 2021; Ajao et al., 2023; Liu et al., 2023). In particular, our research focuses on identifying the relationship between the dietary crude protein concentrations and the severity of *Eimeria* infection. It is well-established that 20.5% to 22% of dietary crude protein is associated with increased severity of signs of coccidiosis, including lethargy, oocyst production, blood in droppings, and mortality (Gomez-Osorio et al., 2021). This phenomenon is linked to increased trypsin and bile secretion by increased dietary crude protein content. Trypsin degrades the oocyst's stieda body, facilitates a more extensive oocyst excystation, and releases the infective sporozoites in greater numbers (Sharma et al., 1973). Further, bile salts stimulate sporozoite motility, exacerbating the infection's severity and clinical manifestations of coccidiosis (Lopez-Osorio et al., 2020; Gomez-Osorio et al., 2021). Moreover, high-protein diets predispose birds to secondary enteric infections, such as necrotic enteritis (Fathima et al., 2022). This dual impact of high protein diets on primary and secondary infections underscores the importance of a balanced dietary approach to mitigate coccidiosis.

Reducing the dietary crude protein levels by up to 3%, below the current recommendations, may not negatively impact the growth performance of broilers, especially when sufficient non-essential nitrogen is provided (Siegert and Rodehutscord, 2019; Olukosi and Lin, 2024). During the *Eimeria* infection, there is a reduction in the ileal digestibility of amino acids (AA) (Amerah and Ravindran, 2015). The supplementation of supplemental AA significantly improves the apparent ileal digestibility of the supplemented AA (Teng et al., 2021), thereby increasing the availability of nutrients to meet the higher maintenance requirements. The increased commercial availability of supplemental AA enables nutritionists to formulate cost-effective RP diets supplemented with essential supplemental AA to meet nutrient requirements. The RP diets supplemented with essential AA can improve the immunity and antioxidant capacity of the birds in their defense against enteric infections (Bortoluzzi et al., 2018). In addition, supplementation of AA with higher digestibility during enteric diseases, when the intestine's absorptive capacity is compromised, proves beneficial in restoring loss in production and enhancing the overall health of the birds (Bortoluzzi et al., 2018; Teng et al., 2021).

Soybean meal is predominantly used as the protein source in poultry diets due to its high protein content and superior AA profile, as well as because there are limitations (both nutritional and antinutritional factors) to using alternative protein feedstuffs. On the other hand, CM and cDDGS are regarded as viable alternative protein feedstuffs in many parts of the world due to their relatively high levels of crude protein (on the average 36% and 25% crude protein, respectively) and digestible AA (Salim et al., 2010; Khajali and Slominski, 2012). Although there are numerous reports on the use of CM and cDDGS in broiler chicken diets, their use in RP diets has not received much attention. Our previous study showed that CM and cDDGS did not support the same level of growth performance when crude protein was lowered by 45 g/kg relative to the standard protein diet in broiler chickens (Ajao et al., 2022). However, to our knowledge, the immune response of broiler chickens infected with coccidiosis and receiving RP diets with SBM, CM, or cDDGS has not been reported previously. Hence, the primary objective of this study was to compare the effects of SBM, CM, and cDDGS inclusion in RP diets for broilers during an *Eimeria* infection using growth performance and immune response criteria.

## 2 Materials and methods

All animal experiment procedures used in the study were approved by the Institutional Animal Care and Use Committee at the University of Georgia (A-21-06-006).

### 2.1 Experimental diets

All the birds received one starter (d 0 to 7) diet formulated to meet the Cobb nutrient and energy recommendation. Four diets were formulated for the grower (d 7 to 28) and finisher (d 28 to 42) phases. The four diets for the grower phase were (i) a standard diet with crude protein at 200 g/kg (SP); (ii) a RP (crude protein at 160 g CP/kg) corn-SBM diet (RP-SBM); (iii) a RP diet in which 80 g/kg CM replaced 60 g/kg SBM (RP-CM); and (iv) a RP diet in which 100 g/kg cDDGS replaced 50 g/kg SBM (RP-cDDGS). All the experimental diets were isocaloric and formulated to have similar digestible AA content (Table 1) according to Cobb 500 recommendation (Cobb, 2021). The starter diet was fed as crumbs, and the experimental diets were fed as pellets.

**Table 1:** Ingredients (g/kg, as-fed basis) and calculated chemical composition (g/kg, dry matter basis) of the starter and experimental grower diets.

Item	Starter	SP	RP-SBM	RP-CM	RP-cDDGS
Ingredients					
Corn	586.3	637.53	732.33	707.81	680.37
Soybean meal	355.9	306.0	204.0	146.0	157.0
Canola meal				80.0	
cDDGS					100.0
Soybean oil	25.0	25.0	12.0	12.0	9.0
Dicalcium phosphate	17.5	8.0	8.65	8.0	6.8
Limestone	5.6	8.2	8.4	7.8	9.8
L-Lys▪HCl	0.72	1.1	4.7	5.3	5.9
DL-Met	2.00	1.5	2.05	1.95	1.98
L-Thr	0.28	0.20	1.85	2.0	2.14
L-Try			0.1	0.2	0.24
L-Val			1.20	1.3	1.42
L-Arg			2.65	3.2	3.7
L-Cys		0.92	1.55	1.55	1.55
L-Gly			2.1	2.1	2.1
L-Ile			0.9	1.27	1.28
L-Ser			2.62	2.62	2.62
Titanium dioxide		3.0	3.0	3.0	3.0
Sodium bicarbonate	2.0	2.0	2.0	2.0	1.45
Salt	2.8	3.5	3.5	3.5	3.2
Vitamin premix^1^	1.0	1.0	1.0	1.0	1.0
Trace mineral premix^2^	0.8	0.8	0.8	0.8	0.8
Potassium carbonate		1.15	4.5	6.5	4.55
Phytase (Quantum Blue)	0.1	0.1	0.1	0.1	0.1
Total	1000	1000	1000	1000	1000
Calculated nutrients and energy					
ME, kcal/kg	3038	2967	2992	2979	2996
Crude protein	221	201	160	160	161
Total Ca	7.23	6.29	6.26	6.32	6.24
Total P	5.51	5.09	4.84	5.13	4.78
Available P^3^	2.99	2.59	2.56	2.51	2.54
dEB, mEq/kg		245	249	247	247
Digestible amino acids					
Arg	14.9	13.3	12.5	12.5	12.5
His	5.95	5.44	4.34	4.24	4.20
Ile	9.58	8.61	7.53	7.56	7.55
Leu	18.8	17.7	14.9	14.3	15.4
Lys	12.7	11.6	11.6	11.6	11.6
Met	4.60	4.70	4.73	4.72	4.72
Cys	3.58	4.22	4.26	4.37	4.22
Phe	10.9	9.92	7.78	7.28	7.57
Thr	8.71	7.83	7.83	7.85	7.81
Trp	2.61	2.31	1.82	1.88	1.81
Val	10.5	9.59	8.81	8.81	8.82
Total sulfur amino acids	8.17	8.92	8.99	9.09	8.93
Phe + Tyr	19.0	17.3	13.6	12.8	13.3
Gly	7.16	2.11	2.12	2.11	2.11
Ser	9.21	8.38	9.23	8.85	9.10
Gly-equivalent	13.7	8.09	8.71	8.43	8.61

SP = standard protein; RP = reduced-protein; SBM = soybean meal; CM = canola meal; cDDGS = corn distillers' dried grains with solubles; dEB = dietary electrolyte balance.

1 Supplemented per kilogram of diets: vitamin A, 5484 IU; vitamin D_3_, 2643 IU; vitamin E, 11 IU; menadione sodium bisulfite, 4.38 mg; riboflavin, 5.49 mg, D-pantothenic acid, 11 mg; niacin, 44.1 mg, choline chloride, 771 mg; vitamin B_12_, 13.2 mg; biotin, 55.2 mg; thiamine mononitrate, 2.2 mg; folic acid, 990 mg; pyridoxine hydrochloride, 3.3 mg.

2 Supplemented per kilogram of diets: iodine, 1.11 mg; manganese, 66.06 mg; copper, 4.44 mg; iron, 44.1 mg; zinc, 44.1 mg; selenium, 300 mg.

3 Available P level excluded the matrix for the phytase.

### 2.2 Birds and housing

On the day of hatch, a total of 1120 Cobb-500 male broiler chicks were randomly distributed into 56 floor pens in an environment-controlled room. On d 7, the chickens were distributed in a randomized complete block design with a 4 × 2 (four diets × with or without *Eimeria* infection) factorial arrangement until d 42. Each pen had 20 chicks on d 7. The eight treatment groups were (i) SP, (ii) RP-SBM, (iii) RP-CM, (iv) RP-cDDGS, (v) SP with *Eimeria* infection (SP +E), (vi) RP-SBM with *Eimeria* infection (RP-SBM +E), (vii) RP-CM with *Eimeria* infection (RP-CM +E), and (viii) RP-cDDGS with *Eimeria* infection (RP-cDDGS +E). All the birds were housed in the same room, but the challenged and unchallenged birds were physically separated by being on different sides of the house. Cross-contamination was minimized as much as possible by ensuring no cross-traffic from the challenged to the unchallenged birds. The unchallenged birds were first attended to during routine husbandry. The chickens had ad libitum access to feed and water throughout the experiment.

### 2.3 *Eimeria* infection

On d 15, birds were orally gavaged with 1 mL mixed species of sporulated *Eimeria* oocysts/bird (12,500 oocysts of *E. maxima*, 12,500 oocysts of *E. tenella*, and 62,500 oocysts of *E. acervulina*) or 1 mL of distilled water. The *Eimeria* sporulated oocysts used were field strain oocysts from North Carolina, and the cloning procedure was previously described (Aggrey et al., 2019; Schneiders et al., 2019, 2020).

### 2.4 Chemical analysis

Diet samples were ground through a 0.5-mm sieve (Retsch ZM 200, Retsch GmbH and Co., KG., Germany). Except stated otherwise, all chemical analyses were done using AOAC methods (AOAC, 2016). Diet samples were analyzed for dry matter using a drying oven (VWR International Radnor, PA 19087, USA) and the AOAC method (method 934.01). The nitrogen content of the sample was analyzed using the combustion method (method 968.06) using a LECO FP 828-MC nitrogen analyzer. Samples for AA analysis were hydrolyzed for 24 h in 6 mol/L hydrochloric acid at 110 °C under an atmosphere of N. For Met and Cys, performic acid oxidation was carried out before acid hydrolysis. The AA in the hydrolysate was determined by HPLC after post-column derivatization [Method 982.30E (a, b, c)]. Table 2 shows the analyzed composition of the experimental grower diets. The analyzed crude protein of the RP diets was greater than the calculated. This is mainly because the N content of the supplemental AA in the RP diet was not considered in the diet formula. The crude protein calculation is based on the total N analyzed, some of which are the components of the individual supplemental AA. The analyzed AA profile represents the total analyzed AA (the diets were formulated on a digestible AA basis).

**Table 2:** Analyzed chemical composition (g/kg, dry matter basis) of the experimental diets.

Item	SP	RP-SBM	RP-CM	RP-cDDGS
Dry matter	893	896	893	892
Nitrogen	224	218	207	202
Essential amino acids (total basis)				
Arg	12.9	12.5	12.6	14.5
His	5.25	4.25	4.58	4.69
Ile	9.16	7.93	8.38	8.60
Leu	17.5	14.6	15.8	16.1
Lys	12.8	12.5	12.8	13.0
Met	4.92	4.80	4.00	4.90
Phe	10.1	7.93	7.38	8.60
Thr	7.71	7.60	8.16	8.72
Trp	2.91	2.23	2.31	2.35
Non-essential amino acids (total basis)				
Ala	10.3	8.83	9.39	9.59
Asp	19.8	14.9	15.3	15.4
Cys	4.36	4.36	4.13	4.82
Glu	36.4	30.3	31.6	31.3
Gly	9.72	9.50	8.90	9.90
Pro	12.1	9.94	11.1	11.9
Ser	8.72	8.83	8.60	9.17
Tyr	7.04	5.59	5.14	5.92

SP = standard protein; RP = reduced-protein; SBM = soybean meal; CM = canola meal; cDDGS = corn distillers' dried grains with solubles.

### 2.5 Growth performance measurement

The birds and feed were weighed per pen on d 0, 7, 14, 18, 21, 28, and 42. The growth performance was divided into different phases to enable the growth performance to be characterized for the various phases of the experiment, as follows. The pre-infection phase (d 7 to 14), pre-patent phase (d 15 to 18), acute phase (d 18 to 21), recovery phase (d 21 to 28), and finisher phase (d 28 to 42). Mortality was recorded as it occurred and used to correct weight gain and feed intake to calculate mortality-corrected FCR.

### 2.6 Cecal tonsil and spleen T cell population during the *Eimeria* infection

All sample collection for immune response (*n* = 7) was done at 6 d post-coccidia infection (21 d of age). Cecal tonsils (CT) and spleen were collected from one randomly selected bird per pen after euthanasia using cervical dislocation. Single-cell suspensions of the CT and spleen were enriched for lymphocytes by density gradient centrifugation over Histopaque (1.077 g/mL, Sigma Aldrich, St. Louis, MO, USA) for 15 min at 400 × *g* as described earlier (Shanmugasundaram et al., 2022). The cells (1 × 10^6^) were incubated with a 1:250 dilution of unlabeled mouse immunoglobulin A (IgA) for 30 min followed by incubation with 1:250 dilution of primary fluorescein isothiocyanate (FITC)-linked mouse anti-chicken CD4 (Clone CT-4; Southern Biotech, Birmingham, AL, USA) and 1:450 dilutions of phycoerythrin-linked mouse anti-chicken CD8 (Clone CT-8; Southern Biotech, Birmingham, AL, USA) for CD4^+^ and CD8^+^ analysis, respectively. For CD4^+^CD25^+^ T-cell analysis, 1 × 10^6^ cells were incubated with 1 μg/mL of mouse anti-chicken CD25 antibody along with unlabeled mouse IgA at 1:100 dilution for 40 min at 4 °C, after which a 1:250 dilution of FITC-conjugated mouse anti-chicken CD4 was added and incubated further for 20 min at 4 °C (Shanmugasundaram and Selvaraj, 2011). After incubation, the cells were washed with an ice-cold wash buffer (1× phosphate buffered saline [PBS] pH 7.4, 3 mmol/L ethylenediaminetetraacetic acid [EDTA], 3% fetal bovine serum [FBS]) to remove unbound antibodies. The percentage of CD4^+^, CD8^+^, and CD4^+^CD25^+^ cells were analyzed using a flow cytometer (Guava EasyCyte, Millipore, Billerica, MA, USA), and the CD8^+^:CD4^+^ ratios were calculated. The percentage of CD4^+^CD25^+^ cells was expressed as a percentage of total CD4^+^ cells (Liu et al., 2023).

### 2.7 Mitogen-stimulated splenocyte proliferation during *Eimeria* infection

The spleen was collected from one bird in each of the seven pens per treatment group (*n* = 7). The splenocytes proliferation efficiency was measured using the 3-[4,5-dimethylthiazol-2-yl]-2,5-diphenyl tetrazolium bromide (MTT) colorimetric assay as described previously (Shanmugasundaram et al., 2019). Briefly, splenocytes were collected, and single-cell suspension was prepared by density gradient centrifugation. Live cells were counted by trypan blue exclusion using an automated cell counter (Thermo Fisher Scientific, Waltham, MA, USA). Splenocytes (1 × 10^6^ cells) were cultured in a 250 μL cell culture medium (RPMI-1640 supplemented with 4% fetal bovine serum, 2% chicken serum, and 1% penicillin and streptomycin) supplemented with 1 μg/mL of concanavalin A (ConA) in 96-well plates incubated for 72 h at 37 °C in the 5% CO_2_ incubator. After 72 h incubation, 20 μL of 5 mg/mL methyl thiazolyl tetrazolium (MTT) (Sigma–Aldrich, St. Louis, MO, USA) solution was added to the cell culture and incubated for an additional 4 h. The supernatant was removed by centrifugation, and cells were resuspended in 200 μL of isopropanol + 10% dimethyl sulfoxide in 0.04 mol/L HCl and allowed to stand for 1 h at room temperature. The concentration of MTT formazan formed in the 96-well plates was read using an ELISA plate reader at 570 nm. Proliferation efficiency was reported as the mean optical density.

### 2.8 Quantification of bile anti-coccidia IgA content during *Eimeria* infection

The bile was collected from one bird in each of the seven replicate pens per treatment group (*n* = 7) and stored at −20 °C until further analysis. Bile anti-coccidia IgA was determined by ELISA as described previously (Markazi et al., 2019; Liu et al., 2023) with minor modification. The optimal dilutions of bile, antigen, and primary and secondary antibodies were standardized using checkerboard titrations. Briefly, flat-bottomed, high-binding 96-well plates were coated with 10 μg/mL of coccidia antigen in 100 μL/well of 0.1 mol/L carbonate buffer (pH 9.6) and incubated overnight at 4 °C. The plates were washed three times using wash buffer (0.05% Tween 20 in PBS, pH 7.4) and blocked with 8% non-fat dried milk in wash buffer (200 μL/well) and incubated for 90 min at 37 °C. After incubation, the plates were washed, and bile samples diluted to 1:100 with blocking buffer were added to the plate (100 μL/well). The plates were incubated for 90 min at 37 °C. The plates were washed three times with wash buffer, and horseradish peroxidase (HRP)-conjugated polyclonal goat anti-chicken IgA (Bethyl Laboratories, Montgomery, TX) diluted to 1:50,000 in 5% non-fat dry milk buffer was added to the plate at 100 μL/well. The plates were incubated at 37 °C for 1 h. After incubation, the plates were washed with PBS-Tween 20, and the substrate 3,3,5,5-tetramethylbenzidine (Sigma–Aldrich, St. Louis, MO, USA) was added at 100 μL/well and incubated for 15 min. The reaction was stopped using 1 N HCl (100 μL/well), and absorbance was measured at 450 nm using a microplate reader (Synergy HTX, multi-mode microplate reader, BioTek Instruments, Inc., VT, USA). The IgA values were reported as mean optical density.

### 2.9 Splenic macrophage nitric oxide production measurement

Macrophages were collected from one bird per pen in each of the seven pens per treatment group (*n* = 7), as described previously (Shanmugasundaram et al., 2019). Briefly, single-cell suspension of the splenocytes was prepared by density gradient centrifugation using Histopaque (1.077 g/mL) for 15 min at 450 × *g* as previously described (Shanmugasundaram and Selvaraj, 2012). The splenocytes (1 × 10^9^) were cultured in a 75-cm^2^ flask in RPMI-1640 medium supplemented with 4% fetal bovine serum, 2% chicken serum, and 1% penicillin and streptomycin using a 5% CO_2_ incubator at 37 °C. After 24 h of incubation, the adherent cells were harvested by trypsinization. Cells were washed and counted using a cell counter (Thermo Fisher Scientific, Lenexa, KS, USA), and 1 × 10^5^ cells were reseeded per well in a 96-well flat-bottom plate along with 10 μg/mL of coccidia antigen and incubated for 48 h at 37 °C and 5% CO_2_ level. At 48 h of culture, the plates were centrifuged at 450 × *g* for 5 min, and the supernatant was removed. The nitrite content of the supernatant was determined using a sulfanilamide/N-(1-Naphthyl) ethylenediamine dihydrochloride solution (#R2233500, Ricca Chemical Company, Arlington, TX, USA). The resultant solution's optical density was read at 550 nm using a microplate ELISA reader (Epoch plate reader, BioTek, VT, USA). A standard curve with 0, 7.825, 15.625, 31.25, 62.5, 125, 250, and 500 μmol/L sodium nitrite was drawn using Gen5 software (Synergy HTX, multi-mode microplate reader, BioTek Instruments, Inc. VT, USA) in the same plate as the samples.

### 2.10 Cecal tonsil and splenic cytokine mRNA amounts during an *Eimeria* infection

At 6-d post-coccidia infection, cecal tonsils and spleen from one bird per replicate (*n* = 7) were collected and fixed in 1.5 mL of RNAlater and stored in a −80 °C freezer until further analysis (Kiba et al., 2007). Total RNA was extracted from the tissues using the TRI Reagent (Molecular Research Center, Cincinnati, OH, USA), following the manufacturer's instruction (Selvaraj and Klasing, 2006). The total RNA was reverse transcribed to cDNA and analyzed for the relative expression of the pro-inflammatory cytokines interferon-gamma (*IFN-γ*), *IL-17*, the anti-inflammatory cytokines *IL-10* and transforming growth factor beta (*TGF-β*), using SYBR Green after normalizing with β-actin to adjust for sample to sample and run to run variation. The following machine settings were used to perform real-time PCR for all genes: an initial denaturation of 95 °C for 3 min (1 cycle), followed by 95 °C for 15 s and 57.5 °C for 45 s (40 cycles). The annealing temperatures were as follows: β-actin, *IFN-γ*, *IL-17*, 57.5 °C; and *TGF-β* and *IL-10*, 59 °C. The melting profile of each sample was analyzed after every PCR run to confirm PCR product specificity. The stability of housekeeping genes β-actin ribosomal protein S13 mRNA and glyceraldehyde-3-phosphate dehydrogenase (*GAPDH*) was analyzed using Normfinder software (Department of Molecular Medicine, Aarhus University Hospital, Denmark) as described previously (Shanmugasundaram et al., 2022). The β-actin had the most stable expression and was selected for data normalization, and the 2^−ΔΔCT^ method was used to determine the expression of targeted genes (the CT was the threshold cycle) where Ct is the threshold cycle. The non-infection group was used as a reference group (Livak and Schmittgen, 2001).

### 2.11 Statistical analysis

All data analyses were performed using JMP Pro 16 software (JMP Statistical Discovery LLC) to examine the main effects (diet and *Eimeria* infection) and their interaction. The data obtained for nitric oxide (NO) assay, gene expression analysis, and flow cytometry were analyzed by two-way ANOVA and were considered significantly different at *P* < 0.05. Tendency was defined as 0.05 ≤ *P* < 0.10. When the main effects were significant, differences between means were analyzed using Tukey's honestly significant difference comparison. Pen was considered the experimental unit for all analyses.

## 3 Results

### 3.1 Growth performance

In the pre-infection phase, birds that received the RP-CM diet were lighter (*P* < 0.01) than the birds that received the SP diet (Table 3). There was no treatment effect on feed intake in the pre-infection phase and no treatment effect on weight gain and feed intake in the prepatent phase. There was diet × *Eimeria* infection on weight gain (*P* = 0.030, Table 3) and feed intake (*P* = 0.033, Table 4) during the acute phase of the infection. In the infected group, birds that received RP-SBM tended (*P* = 0.065) to have greater weight gain than the other treatments during the acute phase of the infection, but there were no treatment differences among the uninfected group. There were no significant treatment effects on weight gain during the recovery and finisher phases. The infected birds had lower body weight on d 28 (*P* < 0.001) and 42 (*P* = 0.001), irrespective of the diet they received.

**Table 3:** Weight gain and feed intake of broiler chickens receiving diets with standard or reduced-protein levels with different protein feedstuffs before, during, and after coccidia infection.^1^^,^^2^^,^^3^^,^^4^

Item	Weight gain, g					Body weight, g		
	Pre-infection	Prepatent	Acute	Recovery	Finisher	Day 8	Day 28	Day 42
SP		303	288^a^	613	1810	200	1692	3503
RP-SBM		296	242^a^	628	1653	201	1641	3294
RP-CM		297	249^a^	625	1604	199	1625	3229
RP-cDDGS		296	251^a^	620	1727	199	1653	3380
SP +E		294	86^c^	585	1764	199	1448	3212
RP-SBM +E		291	125^bc^	582	1692	198	1452	3144
RP-CM +E		288	84^c^	606	1687	200	1440	3129
RP-cDDGS +E		310	68^c^	582	1690	198	1429	3119
Pooled SEM		7.7	14.1	21.9	67.1	0.5	23.7	58.9
Means for the main effect of diets								
SP	285^a^	280	187	604	1787	199	1576	3357
RP-SBM	270^a,b^	270	183	604	1673	199	1545	3219
RP-CM	260^b^	276	166	618	1646	199	1535	3179
RP-cDDGS	278^a,b^	267	160	599	1708	199	1540	3249
Pooled SEM	5.8	6.3	10.5	17.8	49.6	0.4	17.6	43.8
Means for the main effect of *Eimeria* infection								
Uninfected		282	257^a^	624	1699	200	1656^a^	3351^a^
Infected		266	91^b^	589	1708	199	1442^b^	3151^b^
Pooled SEM		5.1	7.8	11.2	31.2	0.3	12.1	30.2
*P*-values								
Diet	0.004	0.658	0.201	0.886	0.239	0.521	0.536	0.074
Infection		0.801	<0.001	0.074	0.602		<0.001	0.001
Diet × *Eimeria* infection		0.463	0.030	0.945	0.714		0.582	0.402

^a^^-c^Means in a column within a group with different superscripts are significantly different (*P* < 0.05).

1 SP: standard protein (200 g/kg crude protein); RP: reduced-protein (160 g/kg crude protein); SBM: soybean meal; CM: canola meal; cDDGS: corn distillers' dried grains with solubles; E: *Eimeria* infection.

2 The pre-infection period was between 7 and 14 d of age. Experimental diets were fed to birds starting from 7 d of age.

3 Pre-patent, acute, recovery, and finisher phases were 0 to 4 d, 4 to 7 d, 7 to 14 d, and 14 to 28 d post-infection, respectively.

4 Birds in the E groups were infected with mixed *Eimeria* oocysts on d 15 of age.

There were no treatment effects on feed intake except the diet × *Eimeria* infection (*P* = 0.033) and the main effect of infection (*P* < 0.001) during the acute phase (Table 4). In the uninfected groups, feed intake tended to be greater (*P* < 0.10) for the birds fed SP compared to other diets, whereas there were no differences in feed intake among the birds in the infected group fed the different diets. There were no treatment effects on FCR at any phase except the main impact of *Eimeria* infection during the acute phase when the FCR of the infected birds was greater (*P* < 0.001) than the uninfected birds.

**Table 4:** The FCR and body weights of broiler chickens receiving diets with standard or reduced-protein levels with different protein feedstuffs before, during, and after coccidia infection.^1^^,^^2^^,^^3^^,^^4^

Item	Feed intake, g					FCR				
	Pre-infection	Prepatent	Acute	Recovery	Finisher	Pre-infection	Prepatent	Acute	Recovery	Finisher
SP		421	377^a^	930	2904		1.40	1.32	1.52	1.63
RP-SBM		408	268^ab^	989	2824		1.38	1.10	1.58	1.72
RP-CM		401	244^ab^	970	2751		1.35	1.00	1.56	1.72
RP-cDDGS		409	273^ab^	1045	2955		1.38	1.09	1.70	1.72
SP +E		401	187^b^	939	2949		1.37	2.30	1.62	1.67
RP-SBM +E		404	205^b^	892	2774		1.39	1.87	1.56	1.65
RP-CM +E		426	207^b^	913	2868		1.50	2.53	1.51	1.70
RP-cDDGS +E		430	217^b^	939	3009		1.39	3.86	1.62	1.80
Pooled SEM		11.3	25.6	35.5	75.0		0.046	0.58	0.076	0.068
Means for the main effect of diets										
SP	453	411	282	962	2926	1.59	1.37	1.81	1.60	1.65
RP-SBM	435	406	227	929	2799	1.61	1.38	1.45	1.55	1.69
RP-CM	431	417	236	985	2809	1.66	1.43	1.76	1.61	1.71
RP-cDDGS	451	421	245	983	2982	1.62	1.39	2.48	1.65	1.76
Pooled SEM	23.7	9.2	20.8	28.9	61.0	0.083	0.038	0.372	0.062	0.055
Means for the main effect of *Eimeria* infection										
Uninfected		413	290^a^	1009	2858		1.38	1.13^b^	1.62	1.70
Infected		415	204^b^	921	2900		1.41	2.64^a^	1.58	1.71
Pooled SEM		7.4	16.8	23.3	38.4		1.37	0.26	0.039	0.035
*P*-values										
Diet	0.896	0.770	0.207	0.498	0.123	0.944	0.821	0.264	0.525	0.573
Infection		0.483	<0.001	0.194	0.158		0.396	<0.001	0.557	0.755
Diet × *Eimeria* infection		0.201	0.033	0.400	0.749		0.306	0.211	0.723	0.806

^a,b^Means in a column within a group with different superscripts are significantly different (*P* < 0.05).

1 SP: standard protein (200 g/kg crude protein); RP: reduced-protein (160 g/kg crude protein); SBM: soybean meal; CM: canola meal; cDDGS: corn distillers' dried grains with solubles; E: *Eimeria* infection.

2 The pre-infection period was between 7 and 14 d of age. Experimental diets were fed to birds starting from 7 d of age.

3 Pre-patent, acute, recovery, and finisher phases were 0 to 4 d, 4 to 7 d, 7 to 14 d, and 14 to 28 d post-infection, respectively.

4 Birds in the E groups were infected with mixed *Eimeria* oocysts on d 15 of age.

### 3.2 Cecal tonsil T cell population

There was no significant diet × *Eimeria* infection interaction on cecal tonsil CD4^+^, CD8^+^, and CD25^+^CD4^+^ cell percentages and CD8^+^:CD4^+^ cell ratio (Table 5). There was a significant (*P* = 0.025) main effect of diet on the cecal tonsil CD8^+^ cell percentage. Birds in the RP-CM group had a significantly higher (*P* < 0.05) CD8^+^ percentage than those in the SP group. There was no significant effect of infection on the CD8^+^ percentage. The *Eimeria* infection significantly increased (*P* < 0.001) the CD4^+^ and CD4^+^CD25^+^ percentages and decreased (*P* < 0.001) the CD8^+^:CD4^+^ cell ratio.

**Table 5:** Cecal tonsil T cell population for broiler chickens receiving diets with standard or reduced-protein levels with different protein feedstuffs during an active *Eimeria* infection (6 d post-infection).^1^^,^^2^

Item	CD8^+^, %	CD4^+^, %	CD8^+^:CD4^+^	CD4^+^CD25^+^, %
SP	23.2	15.8	1.47	5.60
RP-SBM	23.8	16.2	1.47	5.79
RP-CM	24.0	15.9	1.51	5.61
RP-cDDGS	23.3	16.0	1.46	5.61
SP +E	22.9	18.1	1.26	6.91
RP-SBM +E	23.9	17.9	1.34	6.54
RP-CM +E	23.8	17.8	1.33	6.52
RP-cDDGS +E	23.6	18.1	1.30	6.50
Pooled SEM	0.30	0.24	0.022	0.175
Means for the main effect of diets				
SP	23.0^b^	17.0	1.37	6.26
RP-SBM	23.9^ab^	17.0	1.40	6.16
RP-CM	23.9^a^	16.9	1.42	6.07
RP-cDDGS	23.4^ab^	17.1	1.38	6.06
Pooled SEM	0.21	0.17	0.013	0.125
Means for the main effect of *Eimeria* infection				
Uninfected	23.6	16.0^b^	1.48^a^	5.65^b^
Infected	23.5	18.0^a^	1.31^b^	6.62^a^
Pooled SEM	0.15	0.12	0.012	0.085
*P*-values				
Diet	0.025	0.872	0.126	0.610
Infection	0.799	<0.001	<0.001	<0.001
Diet × *Eimeria* infection	0.772	0.572	0.594	0.395

^a,b^Means in a column within a group with different superscripts are significantly different (*P* < 0.05); *n* = 7.

1 SP: standard protein (200 g/kg crude protein); RP: reduced-protein (160 g/kg crude protein); SBM: soybean meal; CM: canola meal; cDDGS: corn distillers' dried grains with solubles; E: *Eimeria* infection.

2 Birds were fed experimental grower diets between 7 and 28 d of age. At 15 d of age, birds were infected (+E) or uninfected with *Eimeria*. Analyses were done at 6 d post-infection.

### 3.3 Cecal tonsil cytokine mRNA amounts

There was a significant diet × *Eimeria* infection (*P* = 0.004) in cecal tonsil *IL-17* mRNA amounts (Table 6). The *IL-17* mRNA amounts were not significantly different among the uninfected birds fed the different diets, whereas, among the infected birds, the *IL-17* mRNA amount was greater (*P* < 0.05) for birds fed RP-SBM compared with all other diets. The mRNA amounts for *IL-10* and *IFN-γ* were higher (*P* < 0.001) for *Eimeria*-infected compared with non-infected groups. There were no treatment effects (*P* = 0.739) on *TGF-β* mRNA amount.

**Table 6:** Cecal tonsil cytokine mRNA amounts for broiler chickens receiving diets with standard or reduced-protein levels with different protein feedstuffs during an active *Eimeria* infection (6 d post-infection).^1^^,^^2^

Item	*IL-10*	*IFN-γ*	*IL-17*	*TGF-β*
SP	1.00	1.00	1.00^cd^	1.00
RP-SBM	0.89	0.99	1.05^cd^	1.58
RP-CM	1.02	0.93	1.31^bcd^	1.42
RP-cDDGS	0.99	1.03	0.86^d^	1.11
SP +E	2.40	2.64	2.01^bcd^	1.44
RP-SBM +E	1.39	2.34	4.25^a^	1.42
RP-CM +E	3.04	2.31	2.34^bc^	1.57
RP-cDDGS +E	2.87	2.56	2.65^b^	1.32
Pooled SEM	0.531	0.354	0.312	0.354
Means for the main effect of diets				
SP	1.70	1.82	1.50^b^	1.22
RP-SBM	1.14	1.66	2.65^a^	1.50
RP-CM	2.03	1.62	1.83^ab^	1.50
RP-cDDGS	1.93	1.79	1.75^b^	1.21
Pooled SEM	0.372	0.240	0.223	0.245
Means for the main effect of *Eimeria* infection				
Uninfected	0.97^b^	0.99^b^	1.05^b^	1.28
Infected	2.43^a^	2.46^a^	2.81^a^	1.44
Pooled SEM	0.232	0.174	0.155	0.176
*P*-values				
Diet	0.361	0.925	0.005	0.739
Infection	<0.001	<0.001	<0.001	0.523
Diet × *Eimeria* infection	0.487	0.973	0.004	0.860

*IL* = interleukin; *IFN-γ* = cytokines interferon-gamma; *TGF-β* = transforming growth factor beta.

^a–d^Means in a column within a group with different superscripts are significantly different (*P* < 0.05); *n* = 7.

1 SP: standard protein (200 g/kg crude protein); RP: reduced-protein (160 g/kg crude protein); SBM: soybean meal; CM: canola meal; cDDGS: corn distillers' dried grains with solubles; E: *Eimeria* infection.

2 Birds were fed experimental grower diets between 7 and 28 d of age. At 15 d of age, birds were infected (+E) or uninfected with *Eimeria*. Analyses were done at 6 d post-infection.

### 3.4 Macrophage nitric oxide production and the splenic T cell population

There was a significant diet × *Eimeria* infection (*P* < 0.001) for splenic macrophage NO production and CD^8+^:CD^4+^ cell ratio (Table 7). Splenic macrophage NO production was not different among the uninfected birds receiving the different diets; however, among the infected birds, those that received RP-SBM or RP-cDDGS had the highest or lowest NO production, respectively. For splenic CD8^+^:CD4^+^ cell ratio, there was no significant difference among the uninfected birds receiving the different diets, whereas infected birds that received RP-SBM had greater (*P* < 0.05) splenic CD^8+^:CD^4+^ cell ratio compared with those received RP-cDDGS. There were no significant treatment effects on splenic CD8^+^, CD4^+^, or CD4^+^CD25^+^ cell percentages.

**Table 7:** Splenic macrophage nitric oxide (NO) production and T cell population in broiler chickens receiving diets with standard or reduced-protein levels and different protein feedstuffs during an active *Eimeria* infection (6 d post-infection).^1^, ^2^

Item	NO, μg/mL	CD8^+^, %	CD4^+^, %	CD8^+^:CD4^+^	CD4^+^CD25^+^, %
SP	1.26^cd^	24.2	15.9	1.55^ab^	3.70
RP-SBM	1.23^d^	23.7	15.2	1.57^ab^	3.66
RP-CM	1.19^d^	25.2	15.2	1.67^ab^	3.71
RP-cDDGS	1.18^d^	25.1	15.0	1.68^a^	3.83
SP +E	2.67^b^	24.2	16.3	1.49^ab^	3.72
RP-SBM +E	3.35^a^	25.0	14.8	1.69^a^	3.71
RP-CM +E	2.87^ab^	23.9	15.5	1.55^ab^	3.79
RP-cDDGS +E	1.83^c^	22.1	16.5	1.34^b^	3.91
Pooled SEM	0.133	0.30	0.24	0.015	0.171
Means for the main effect of diets					
SP	1.97^b^	24.2	16.1	1.52	3.71
RP-SBM	2.29^a^	24.3	15.0	1.63	3.68
RP-CM	2.03^ab^	24.5	15.4	1.61	3.75
RP-cDDGS	1.51^b^	23.6	15.8	1.51	3.87
Pooled SEM	0.088	0.21	0.17	0.008	0.116
Means for the main effect of *Eimeria* infection					
Uninfected	1.21^b^	24.6	15.3	1.62	3.72
Infected	2.68^a^	23.8	15.8	1.52	3.78
Pooled SEM	0.056	0.15	0.12	0.006	0.076
*P*-values					
Diet	<0.001	0.730	0.188	0.265	0.655
Infection	<0.001	0.227	0.205	0.067	0.603
Diet × *Eimeria* infection	<0.001	0.120	0.329	0.029	0.998

^a–d^Means in a column within a group with different superscripts are significantly different (*P* < 0.05); *n* = 7.

1 SP: standard protein (200 g/kg crude protein); RP: reduced-protein (160 g/kg crude protein); SBM: soybean meal; CM: canola meal; cDDGS: corn distillers' dried grains with solubles; E: *Eimeria* infection.

2 Birds were fed experimental grower diets between 7 and 28 d of age. At 15 d of age, birds were infected (+E) or uninfected with *Eimeria*. Analyses were done at 6 d post-infection.

### 3.5 Splenic cytokine mRNA amounts

There was significant diet × *Eimeria* infection (*P* < 0.001) on spleen *IL-17* mRNA amounts (Table 8). The *IL-17* mRNA amounts were not significantly different among the uninfected birds fed the different diets, whereas, among the infected birds, the *IL-17* mRNA amount was greater (*P* < 0.05) for birds fed RP-SBM compared with all other diets. There were no treatment effects on *IL-10*, *IFN-γ*, and *TGF-β* mRNA amount.

**Table 8:** Splenic cytokine mRNA amounts for broiler chickens receiving diets with standard or reduced-protein levels with different protein feedstuffs during an active *Eimeria* infection (6 d post-infection).^1^, ^2^

Item	*IL-10*	*IFN-γ*	*IL-17*	*TGF-β*
SP	1.00	1.00	1.00^b^	1.00
RP-SBM	0.79	0.82	1.16^b^	1.15
RP-CM	1.14	1.12	1.84^b^	1.16
RP-cDDGS	1.07	1.17	1.51^b^	1.21
SP +E	1.09	1.27	2.32^b^	1.15
RP-SBM +E	0.73	0.98	5.28^a^	0.95
RP-CM +E	1.42	1.58	1.82^b^	1.04
RP-cDDGS +E	1.03	0.81	2.17^b^	1.11
Pooled SEM	0.257	0.293	0.296	0.216
Means for the main effect of diets				
SP	1.05	1.14	1.66^b^	1.08
RP-SBM	0.76	0.90	3.22^a^	1.05
RP-CM	1.28	1.35	1.83^b^	1.10
RP-cDDGS	1.05	0.99	1.84^b^	1.16
Pooled SEM	0.182	0.207	0.209	0.153
Means for the main effect of *Eimeria* infection				
Uninfected	1.00	1.03	1.38^b^	1.13
Infected	1.07	1.16	2.89^a^	1.06
Pooled SEM	0.128	0.146	0.148	0.108
*P*-values				
Diet	0.263	0.437	<0.001	0.958
Infection	0.712	0.515	<0.001	0.664
Diet × *Eimeria* infection	0.901	0.553	<0.001	0.862

*IL* = interleukin; *IFN-γ* = cytokines interferon-gamma; *TGF-β* = transforming growth factor beta.

^a,b^Means in a column within a group with different superscripts are significantly different (*P* < 0.05); *n* = 7.

1 SP: standard protein (200 g/kg crude protein); RP: reduced-protein (160 g/kg crude protein); SBM: soybean meal; CM: canola meal; cDDGS: corn distillers' dried grains with solubles; E: *Eimeria* infection.

2 Birds were fed experimental grower diets between 7 and 28 d of age. At 15 d of age, birds were infected (+E) or uninfected with *Eimeria*. Analyses were done at 6 d post-infection.

### 3.6 Bile anti-coccidia IgA content and mitogen-stimulated splenocytes proliferation

There was a significant diet × *Eimeria* infection interaction on *Eimeria*-specific bile IgA (*P* = 0.015) and conA (*P* = 0.002) responses (Table 9). The interaction for bile IgA is shown by no differences in bile concentration of *Eimeria*-specific bile IgA for the uninfected birds but for the infected birds, birds that were fed RP-SBM had higher (*P* < 0.05) bile IgA than those fed SP or SP-cDDGS. For the ConA (mitogen-stimulated splenocyte proliferation) response, in uninfected birds, ConA response was higher (*P* < 0.05) in birds fed SP compared to all other diets. In contrast, there was no difference in ConA response among the infected birds that received the different diets.

**Table 9:** Bile anti-coccidia immunoglobulin A (IgA) content and mitogen-stimulated splenocyte proliferation for broiler chickens receiving diets with standard or reduced-protein levels with different protein feedstuffs during an active *Eimeria* infection (6 d post-infection).^1^^,^^2^^,^^3^

Item	Bile IgA	Concanavalin A response
SP	0.160^c^	0.640^a^
RP-SBM	0.153^c^	0.392^b^
RP-CM	0.145^c^	0.373^b^
RP-cDDGS	0.165^c^	0.350^b^
SP +E	0.261^b^	0.355^b^
RP-SBM +E	0.380^a^	0.306^b^
RP-CM +E	0.300^ab^	0.395^b^
RP-cDDGS +E	0.274^b^	0.356^b^
Pooled SEM	0.0206	0.0404
Means for the main effect of diets		
SP	0.210^b^	0.497^a^
RP-SBM	0.267^a^	0.349^b^
RP-CM	0.222^ab^	0.384^b^
RP-cDDGS	0.219^ab^	0.353^b^
Pooled SEM	0.0146	0.0279
Means for the main effect of *Eimeria* infection		
Uninfected	0.156^b^	0.439^a^
Infected	0.304^a^	0.353^b^
Pooled SEM	0.0103	0.0204
*P*-values		
Diet	0.040	0.002
Infection	<0.001	0.005
Diet × *Eimeria* infection	0.015	0.002

^a–c^Means in a column within a group with different superscripts are significantly different (*P* < 0.05); *n* = 7.

1 SP: standard protein (200 g/kg crude protein); RP: reduced-protein (160 g/kg crude protein); SBM: soybean meal; CM: canola meal; cDDGS: corn distillers' dried grains with solubles; E: *Eimeria* infection.

2 Birds were fed experimental grower diets between 7 and 28 d of age. At 15 d of age, birds were infected (+E) or uninfected with *Eimeria*. Analyses were done at 6 d post-infection.

3 Means represents the optical density (OD) values.

## 4 Discussion

We previously observed superior growth performance in broiler chickens that received an RP diet with SBM compared to those with CM or cDDGS (Ajao et al., 2022). However, the effect of such diets on the immune response has not been explored. Hence, this study aimed to evaluate the impact of RP diets on the immune response of broilers during the *Eimeria* infection. In this experiment, all diets were isocaloric, but the RP diets were formulated to have 4% lower crude protein than the SP diet. All the diets were formulated to the same level of standardized digestible essential AA. Cysteine was added to the RP diets to ensure the same level of digestible total sulfur-AA in all diets without increasing the methionine level. Additionally, glycine and serine were added as sources of non-essential N in the RP diets to ensure adequate synthesis of non-essential AA. The formulated total Ca and non-phytate P contents were reduced to account for additional Ca and P expected from the phytase supplementation. The experimental diets were formulated on a digestible AA basis. However, the analyzed AA profiles of the diets were on a total AA basis. It is essential to note the limitation of comparing digestible and total AA profiles because the similarity in the profile of the former does not necessarily imply the same in the latter.

Attempt was made to ensure similar levels of standardized digestible AA in all the RP diets, but partially replacing SBM with CM or cDDGS was necessarily attended by increases in the fiber component of the resultant diets. Specifically, ADF profile was in the order: RP-cDDGS > RP-CM > RP-SBM and NDF profile was RP-CM > RP-cDDGS > RP-SBM. Alteration in dietary components have been shown to influence immune and phenotypic responses to *Eimeria* challenge both positively and negatively (Wils-Plotz et al., 2013; Fries-Craft et al., 2023). The changes in dietary fiber content and component as observed in the current experiment is an unavoidable consequence of altering feedstuff combinations in diets and these have implications on the immune response as we subsequently expound.

Dividing the growth performance response to different phases enables the study of the effect of diet at these phases, which is essential to understanding how the different diets influence growth performance at every stage of the disease infection. In the pre-infection phase, the birds that received SP diets had greater or marginally greater weight gain than the other treatments. Although this phase was only 7 d in length, the reduction in weight gain due to consuming RP diets is consistent with other studies (Bregendahl et al., 2002; Barekatain et al., 2019; Hilliar et al., 2020) and could be partly due to the higher sensitivity of young birds to dietary manipulations (Lilburn and Loeffler, 2015). The marginal differences in weight gain due to part-replacement of SBM with CM in the RP diets are similar to our previous observation (Ajao et al., 2022). The weight gain effect was not driven by feed intake response because there were no treatment effects on feed intake during the pre-infection phase.

The effect of partly replacing SBM with CM or cDDGS during the disease infection phase, and phases after that are of interest in the current study. The drastic reduction in weight gain and feed intake during the acute phase of the infection confirmed that the *Eimeria* infection was successful. Therefore, the immune responses due to treatments could be validated. Although the margin of difference in weight gain during the acute phase was much greater than marginal differences in feed intake during that phase, the decrease in feed intake largely accounts for the weight gain differences. This observation in weight gain and feed intake during the acute phase of infection, pathogen-induced anorexia, is widely reported in different infection models (Kyriazakis, 2014; van Niekerk et al., 2016; Taylor et al., 2022) and appears to be a survival mechanism for the host during infection possibly by “starving” the infection agent (van Niekerk et al., 2016; Hite et al., 2020).

Although there were no significant treatment effects on weight gain and feed intake during the recovery and finisher phases, the infected birds had lower body weight at the end of the recovery (d 28) and finisher (d 42) phases. This indicates that the infected birds could not fully compensate for the weight loss that occured during the acute infection phase. The dependence of body weight on protein feedstuff was of interest in the current experiment. There was a tendency for a diet effect on d 42 body weight. Although there were numerical differences in the final body weight of birds that received RP diets with different protein feedstuffs, the marginal statistical effect observed was mainly driven by dietary protein levels rather than protein feedstuff used in the RP diets. The same response pattern was observed in the d 42 body weight of the uninfected birds. This strengthened the proposition that the differences in final body weight were mainly influenced by dietary protein content. This agrees with other observations of dietary protein content's effect on broiler chickens' growth performance (Barekatain et al., 2019; Hilliar et al., 2020; Ajao et al., 2022).

Among the infected group during the acute phase of the infection, the RP-SBM group tended to have greater weight gain than all the other treatments in this order: RP-SBM > SP > RP-CM > RP-cDDGS. These treatment differences in the infected group were not as evident during the recovery and finisher phases. Still, the birds in the RP-SBM group had numerically greater body weight on d 42 compared to the other RP groups. Although this effect was not totally accounted for by feed intake, the FCR of the birds in the RP-SBM group was generally lower than for other groups. As the subsequent discussion shows, likely, the marginally higher weight gain of the RP-SBM compared to the other groups during the acute phase was driven by the differences in immune response in the different groups, especially because the immune response analyses were done during the acute phase of the infection.

The *Eimeria* infection decreased gut integrity, mitogen-stimulated splenocyte proliferation, and macrophage NO production but increased cecal tonsil CD4^+^CD25^+^ percentage, splenic *IL-17* mRNA transcription, cecal tonsil *IL-10*, *IFN-γ*, and *IL-17* mRNA transcription, and increase bile anti-*Eimeria* IgA concentration. These observations of the *Eimeria* infection effects align with findings from previous studies (Dalloul et al., 2003; Hong et al., 2006; Teng et al., 2021; Liu et al., 2023).

During an *Eimeria* infection, inflammatory cytokines like *IFN-γ* trigger the expression of inducible nitric oxide synthase (iNOS) in macrophages, resulting in increased NO production (Laurent et al., 2001). Nitric oxide is a crucial component of the defense mechanism against invading pathogens involving reactive nitrogen species. The increased NO production in the *Eimeria*-infected group in the current study was independent of dietary protein content. This suggests that reducing the crude protein content, as done in the current study, did not hinder NO production following *Eimeria* infections.

The NO production was in this order among the *Eimeria*-infected birds: RP-SBM > SP > RP-CM > RP-cDDGS. Likely, this pattern is partly related to the greater digestibility and availability of AA in SBM compared to CM and cDDGS (Adedokun et al., 2006; Kim et al., 2012; Barua et al., 2020). Arginine serves as the precursor for NO production (Hassan et al., 2021), and the increased availability of AA in SBM compared to CM and cDDGS may explain the increased NO production in the RP-SBM +E group. Thus, the partial replacement of SBM with CM or cDDGS in grower diets that contain 4% lower crude protein relative to the standard diet may have a negative impact on the host's immune response to *Eimeria*.

In this present study, *Eimeria* infection led to an increase in *IL-17* mRNA levels in both the cecal tonsils and spleen, simultaneously reducing the *IL-10* mRNA in the cecal tonsils. IL-17 is a pro-inflammatory cytokine that recruits neutrophils and macrophages to inflammation sites and helps maintain gut integrity (Min et al., 2013). The increased levels of *IL-17* were observed in the RP-SBM +E group, with significantly lower *IL-17* mRNA levels in the RP-CM +E and RP-cDDGS +E groups compared to the RP-SBM +E group. *Eimeria* infections typically lead to an increase in *IL-10* production, which suppresses the secretion of pro-inflammatory cytokines and promotes the differentiation of regulatory T-cell subsets in the intestine, ultimately aiding the *Eimeria* in escaping the host's immune response (Rothwell et al., 2004). Even though there was no statistical significance, it is noteworthy that birds in the RP-SBM group had the lowest *IL-10* mRNA levels compared to the SP +E, RP-CM +E, and RP-cDDGS +E groups. However, partial replacement of SBM with CM and cDDGS led to decreased *IL-17* mRNA and increased *IL-10* mRNA production, suggesting an impaired host's immune response against coccidial infection. It is important to note that even though all diets in the experiment were formulated to have similar digestible AA content, the replacement of SBM with CM and cDDGS in RP diets may have altered the balance of digestible essential and non-essential AA due to differences in the chemical profiles of these protein feedstuffs. This altered balance likely contributed to the impaired immune response observed in the RP-CM +E and RP-cDDGS +E groups.

In poultry, the immune response to coccidiosis is a complex interplay of immune components. Although the cell-mediated immune response is the primary mechanism, antibodies contribute significantly to the protective immune response against coccidiosis (Guzman et al., 2003). Antibodies, specifically IgA, play a critical role in defending against pathogens in the gut mucosa. In the present study, among the birds infected with *Eimeria*, those in the RP-SBM +E group had an increased level of anti-coccidial IgA in the bile. This suggests that reducing the crude protein content from 20% to 16% did not impair the host's IgA response to *Eimeria* infection. This dietary change appeared to enhance the production of specific antibodies that can aid in combating coccidial infection. However, further reductions in SBM content, achieved with partial replacement of SBM with cDDGS and CM inclusion, numerically decreased the IgA content, suggesting that the partial replacement of SBM with these feedstuffs in RP diets might compromise the antibody response to coccidial infection.

IFN-γ is a proinflammatory cytokine secreted by the intestinal intraepithelial lymphocytes and contributes significantly to the clearance of *Eimeria* infection in poultry (Hong et al., 2006). Its multifaceted functions, including inhibiting parasitic development, activating antibody-dependent cell-mediated cytotoxicity, and generating free radicals, all aid in combating the infection (Yun et al., 2000). In the present study, the *Eimeria* infection increased the *IFN-γ* mRNA level in the cecal tonsils. However, dietary treatments had no significant effect on the *IFN-γ* expression, suggesting that reducing the crude protein content of the grower diets did not impair the host's IFN-γ production. Thus, the host's ability to produce this proinflammatory cytokine, which plays a pivotal role in fighting *Eimeria* infections, remained robust even with dietary modifications.

The immune response to coccidiosis in poultry is a finely tuned orchestration of various immune components, including antibodies and proinflammatory cytokines like IFN-γ. Activated T cells play a pivotal role in the immune response by undergoing rapid clonal expansion. The lymphocyte proliferation assay is a valuable tool used to assess the ability of T cells to multiply in response to various stimuli, serving as a functional indicator, particularly post-infection (Dalloul et al., 2002). In the present study, it was observed that mitogen-stimulated lymphocyte proliferation was significantly decreased in the RP diet groups, suggesting that the potential mechanism of decreased T cell proliferation is through infection-mediated increase in AA catabolizing enzymes. These enzymes, including inducible iNOS, arginase, indoleamine 2,3 dioxygenase, tryptophan 2,3 dioxygenase, and IL-4-induced gene-1, are known to be upregulated in response to infections. Their role is to catabolize AA, which can have downstream effects on T-cell function.

One such effect is diminished T cell receptor signaling, influencing the activation of T cells. Secondly, there could be decreased activation of the JAK3/STAT5 signaling pathway, which plays a vital role in T cell development and function. Lastly, the interaction between T cells and B cells could be impacted, with a negative effect on the production of antibodies and a stymying of effective immune response (Castellano and Molinier-Frenkel, 2020). Additionally, during periods of stress, such as infections, the nitrogen requirements of birds might be increased. This means the demand for AA could be elevated during an infection. As observed in this study, the use of a RP diet might indirectly interfere with T cell proliferation and the synthesis of immune mediators by limiting the availability of essential AA required for these processes (Castellano and Molinier-Frenkel, 2020).

## 5 Conclusion

In conclusion, this study highlights the intricate relationship between dietary composition and the host's immune response. Although reducing crude protein content in the diet enhanced the IgA response, partial replacement of SBM with CM and cDDGS in the RP diet may adversely affect antibody production. However, diet modifications did not compromise the production of IFN-γ, a critical factor in combating coccidial infections. The observed decrease in mitogen-stimulated lymphocyte proliferation in the RP diet groups suggests that dietary modifications impacting protein content could influence the immune response at the cellular level. These findings emphasize the relationship between dietary factors, immune responses, and the metabolic demands of the immune system during infections. It can be concluded that reducing the crude protein content of the grower diet during the grower period, which was achieved by lowering the SBM content of the diet, did not impair the host immune response to coccidial infection. However, partial replacement of SBM with CM and cDDGS in the RP diet impaired the host immune response, making it inadvisable to completely replace SBM with CM or cDDGS as protein feedstuffs in RP diets.

## Credit Author Statement

**Oluyinka Olukosi:** Conceptualization, Investigation, Data curation, Software, Writing – review & editing, Funding acquisition, Supervision. **Revathi Shanmugasundaram:** Methodology, Software, Data curation, Writing – review & editing. **Adeleye Ajao:** Methodology, Software, Writing – original draft. **Shahna Fathima:** Methodology, Writing – original draft. **Adelumola Oladeinde:** Methodology, Supervision, Writing – review & editing. **Ramesh Selvaraj:** Methodology, Supervision, Writing – review & editing. **Todd Applegate:** Validation, Writing – review & editing.

## Declaration of competing interest

We declare that we have no financial and personal relationships with other people or organizations that can inappropriately influence our work, and there is no professional or other personal interest of any nature or kind in any product, service and/or company that could be construed as influencing the content of this paper.
